# Pulmonary Talcosis Due to Daily Inhalation of Talc Powder

**DOI:** 10.5334/jbsr.1384

**Published:** 2018-01-31

**Authors:** Guillaume Verlynde, Emmanuel Agneessens, Jean-Louis Dargent

**Affiliations:** 1 Department of Radiology, Clinique Saint-Pierre Ottignies, BE; 2 Institute of Pathology and Genetics, Gosselies, BE

**Keywords:** Pulmonary talcosis, Centrilobular nodules, Computed tomography

## Case

A 31-year-old nonsmoking woman, complained of dyspnea and polyarthralgia following a cesarian section. She had no fever, no sputum production, and no cough. The patient worked as domestic help. Physical examination was normal, without auscultation abnormalities. Bloods tests showed D-dimer elevation and a slight hypereosinophilia (780/mm³).

**Figure 1:**
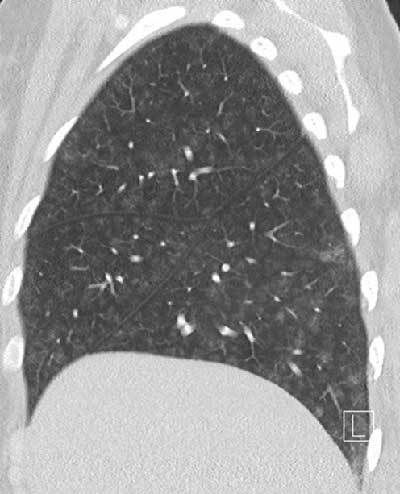
Diffuse groundglass centrilobular nodules without tree-in-bud.

**Figure 2:**
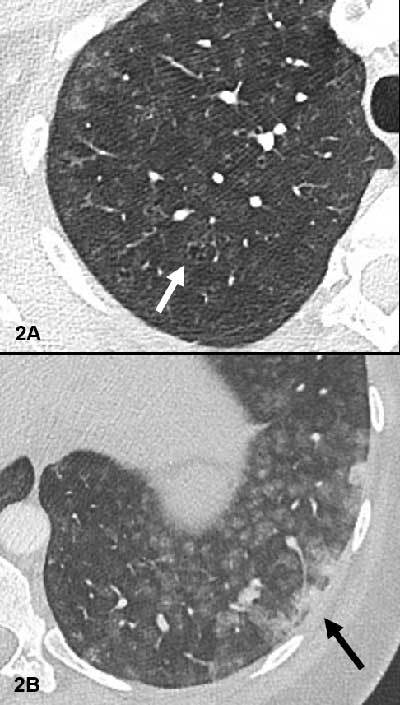
Small centrilobular apical emphysema (white arrow Figure 2A) and confluent condensed areas in the basal segments of the lower lobes (black arrow Figure 2B).

A CT angiogram revealed no evidence of pulmonary embolism. However, diffuse groundglass centrilobular nodules without tree-in-bud pattern were observed (Figure 1), in association with small centrilobular apical emphysema (white arrow Figure 2A) and confluent condensed areas in the basal segments of the lower lobes (black arrow Figure 2B). The first diagnostic hypothesis was hypersensitivity pneumonitis, though there was no air-trapping on the CT. The etiological investigation failed to find any causative agent for this pathology.

**Figure 3:**
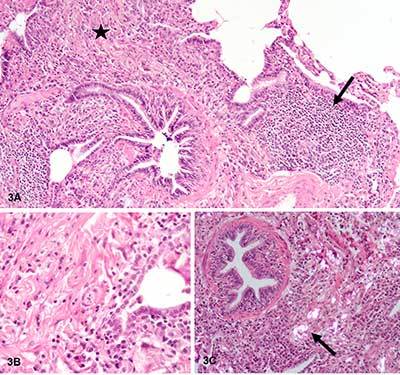
Important non-caseating granulomatous interstitial inflammation, with lymphocytes (black arrow Figure 3A), numerous macrophages and multinuclear giant cells, sitting preferentially in peribronchiolar regions (black star Figure 3A, B). The granulomas contain characteristic needle-shaped birefringent crystalline material in polarized light (black arrow Figure 3C).

Cytology of bronchial alveolar lavage (BAL) showed 16% of neutrophils, 29% of lymphocytes, and 55% of macrophages. There were no eosinophils found. A surgical pulmonary biopsy was performed in order to assess the interstitial pathology. Microscopic examination revealed an important non-caseating granulomatous interstitial inflammation, with lymphocytes (black arrow Figure 3A), numerous macrophages and multinuclear giant cells, sitting preferentially in the peribronchiolar regions (black star Figure 3A, B). These granulomas contain characteristic needle-shaped birefringent crystalline material in polarized light (black arrow Figure 3C). The morphological aspect of these crystals are similar to that of the talc.

A second patient history was carried out. The patient admitted to using abundant cosmetic talcum powder in order to soften the skin daily over several years. She denied any intravenous drug abuse. Based on the patient’s history and the clinical, radiological, and histological findings, the diagnosis of talc induced interstitial lung disease (talcosis) was made based on massive use of talc powder.

## Discussion

Talc (hydrate magnesium silicate) is a mineral widely used in various industrial process and in daily life. Cosmetically, talc can be found in antiperspirants or body powder.

Pulmonary talcosis is usually divided in four types. Talco-silicosis and talco-asbestosis affect miners or industrial workers exposed to a high amount of impure talc dust containing either silica or asbestiform fibers. The third form occurs in the setting of intravenous drug addicts who inject tablets intended for oral use; it’s related to talc pulmonary embolism. The last, the least-known type, is associated with inhalation of pure talc, often due to abundant daily use.

CT findings in a patient with talcosis include diffuse centrilobular nodules, ground-glass opacities, confluent masses with internal foci of high attenuation that are consistent with talc deposition, and emphysema. This centrilobular distribution of the micronodules is suggestive of bronchiolar or vascular involvement. In talcosis associated with use of intravenous drugs, the lung apices and costophrenic sulci are generally spared. Furthermore, if emphysema is present, it is typically centrilobular or apical in talcosis by inhalation while it is panlobular with a basal predominance in the intravenous form [1]. The CT pattern in our case orientates towards an inhalative exposure.

Whether inhaled or injected, talc causes non-necroziting granulomatous inflammation that leads to progressive fibrosis. These granulomas are composed of multinucleated giant cells surrounded by a small amount of fibrous tissue. The definitive diagnosis is made by light microscopic examination of lung tissue specimen, obtained by trans-bronchial or open lung biopsy. Talc can be identified as irregular, birefringent needle-shaped crystals inside or outside macrophages under polarized light. The inflammation is organized around bronchioles and small airways in inhaled forms whereas is perivascular in haematogenous spread. The topography around of bronchioles of the granuloma in the biopsies in our case make intravenous form unlikely.

The natural history of pulmonary talcosis is said to be slowly progressive, even after exposure has ceased. There is no specific treatment.

In conclusion, non-occupational inhalation of talc is a rare cause of talcosis. This case highlights the importance of a detailed medical history with special focus on environmental exposures. It reports suggestive CT patterns to differentiate inhaled and intravenous forms of lung talcosis.
